# Assessment of the Efficacy of the Google Gemini 2.5 Pro Model in Solving the Polish State Specialization Exam in Pediatric Surgery

**DOI:** 10.7759/cureus.95581

**Published:** 2025-10-28

**Authors:** Dawid Boczkowski, Tomasz Dolata, Dominika Radej, Piotr Sawina, Rania Suleiman, Ada Latkowska, Anna Kowalczyk, Michalina Loson-Kawalec, Wojciech Jaworski, Aleksandra Wielochowska, Melania Olender, Aleksandra Latkowska, Patrycja Dadynska, Weronika Majchrowicz, Aleksandra Stachowicz

**Affiliations:** 1 Department of Internal Medicine, Central Teaching Hospital of the Medical University of Lodz, Lodz, POL; 2 Department of Internal Medicine, Multi-specialty Independent Public Healthcare Facility Hospital, Nowa Sól, POL; 3 Department of General and Vascular Surgery, Specialist Medical Center, Polanica-Zdrój, POL; 4 Department of Internal Medicine, Non-Public Health Care Institution (NZOZ) Hospital, Dzierżoniów, POL; 5 Faculty of Medicine, Wrocław Medical University, Wrocław, POL; 6 Faculty of Medicine, University of Opole, Opole, POL; 7 Department of Internal Medicine, Independent Public Provincial Integrated Hospital in Szczecin, Szczecin, POL; 8 Department of Medicine, Central Teaching Hospital of the Medical University of Lodz, Lodz, POL; 9 Faculty of Medicine, Wroclaw Medical University, Wrocław, POL; 10 Faculty of Medicine, Medical University of Silesia, Katowice, POL; 11 Department of Internal Medicine, University Hospital of Karol Marcinkowski, Zielona Góra, POL

**Keywords:** anthropic's claude, artificial intelligence (ai), chat gpt, general pediatric surgery, google gemini

## Abstract

Background

AI language models such as Google Gemini, OpenAI ChatGPT, and Anthropic’s Claude are developing rapidly in response to the growing demand from various sectors of daily life, science, and industry. By collecting and processing extensive datasets, including medical data, they are becoming increasingly popular tools supporting not only IT specialists and programmers but also students and resident physicians in their studies and preparation for examinations, including specialization exams. Consequently, the reliability and accuracy of the information provided by these tools, i.e., AI language models, are often questioned. This concern formed the basis of the present study, which verified the utility of the Google Gemini 2.5 Pro model using the Polish State Specialization Examination (PES) in Pediatric Surgery.

Objective

The objective of this study was to assess the effectiveness and confidence levels of the Gemini 2.5 Pro model in answering PES questions, thereby evaluating its potential educational utility in the specialized surgical field of pediatric surgery.

Methods

The study was conducted using the most recent official PES from the spring 2025 session in pediatric surgery. The exam consisted of 120 multiple-choice questions (five options each, one correct answer). Based on previously published studies and the nature of the questions used in the PES across various medical disciplines in Poland, the questions were divided into two categories: clinical and general (theoretical). Before conducting the test, the Gemini 2.5 Pro model was presented with the PES regulations and then introduced to the examination paper containing the questions in Polish. The correctness of the solved test was verified against the official answer key from the Center for Medical Examinations (CEM) in Łódź. Additionally, the AI model was instructed to rate its confidence in each answer on a five-point scale (from 1 = no confidence to 5 = full confidence). The data obtained were analyzed statistically using the chi-squared test and the Mann-Whitney U test.

Results

The Google Gemini 2.5 Pro model achieved 103 correct answers, corresponding to an overall effectiveness of 85.83%, which is well above the 60% passing threshold. For subgroup analysis, the questions were divided into clinical and general categories, with the model scoring 83% and 91% correct answers, respectively. This difference was not statistically significant (p = 0.417), and the effect size (Cohen’s h = 0.19) indicated a small effect. Furthermore, the model’s confidence ratings showed that correct answers were generally given with higher confidence, while incorrect ones were associated with lower confidence. This suggests a positive correlation between confidence and accuracy, particularly for general questions. However, due to limited data, the exact effect size of this relationship could not be determined.

Conclusions

Gemini 2.5 Pro’s strong performance on the PES demonstrates the considerable potential of advanced AI models in supporting medical education, even in highly specialized fields such as pediatric surgery. The observed association between correctness and declared confidence may help users gauge the reliability of AI-generated responses. Nevertheless, high performance in an examination setting does not eliminate the need for verification and critical evaluation of AI-generated answers in real-world clinical and educational applications.

## Introduction

Modern language models (Large Language Models, LLMs) based on artificial intelligence, such as Google’s Gemini, have become ubiquitous tools that are dynamically revolutionizing numerous sectors, including science and education. The ability of these systems to process and generate natural language at a near-human level opens up new possibilities but also poses challenges regarding the reliability and accuracy of the information they provide.

The Gemini model was first introduced by Google in December 2023. Since then, it has undergone rapid development, leading to more advanced versions such as Gemini 1.5 Pro and the latest Gemini 2.5 Pro [1-2]. Its fast growth and improving capabilities have contributed to a sharp rise in popularity, with over 100 million active users joining the platform within just a few months of its release [2].

A particular area of interest has been the application of LLMs in medical education [3]. The ability of these models to analyze vast amounts of medical data makes them potentially useful in the process of training doctors and preparing them for specialization exams. At the same time, in the medical environment, where the precision and reliability of information are crucial, there are justified doubts about their dependability. In response to these challenges, studies are increasingly being conducted to verify the effectiveness of language models under controlled conditions, such as standardized medical exams [4-13]. These analyses allow for an objective assessment of their “knowledge” and clinical reasoning abilities [3]. Previous studies have shown that leading models, such as GPT-4 or Med-PaLM 2, are capable of achieving high scores on the United States Medical Licensing Examination (USMLE) [7,9,12,14]. The growing research interest in this area creates a need for the systematic verification of subsequent AI generations, especially in the context of local and national examination systems.

The aim of this study was to evaluate the effectiveness and declared self-confidence of the latest version of Google’s language model, Gemini 2.5 Pro, in solving the Polish State Specialization Examination (PES) in the field of pediatric surgery. The analysis was based on the examination paper from the spring 2025 session, consisting of 120 multiple-choice questions. The correctness of the answers provided by the model was verified using the official key issued by the Center for Medical Examinations (CEM) [15]. Additionally, the model was asked to rate its confidence for each answer on a five-point scale, allowing for analysis of the correlation between declared confidence and actual correctness.

## Materials and methods

The main research tool in this analysis, conducted from August 20, 2025, to August 24, 2025, was the Google Gemini language model, version 2.5 Pro. The material for evaluation was the State Specialization Examination (PES) in pediatric surgery from the spring 2025 session. The examination paper, obtained from the official resources of the CEM in Łódź, consisted of 120 single-choice test questions, each with five possible options, only one of which was correct [15]. All interactions with the AI model were conducted in Polish to ensure consistency with the language of the original exam. Each exam question was preceded by a uniform prompt instructing the model to choose the single best answer from the five options provided, without requesting additional explanations, in order to simulate examination conditions as closely as possible. The prompt structure was not altered during the experiment [16].

Following the example of previous similar studies, all examination questions were categorized by the researchers in accordance with previously published studies into two groups: clinical questions, which related to specific cases and medical scenarios, and general questions, which verified theoretical knowledge. Before starting the actual test, the Gemini 2.5 Pro model was instructed on the rules of the PES exam, including the format of the questions and the structure of the test. The collected answers were then verified for correctness based on the official answer key published by the CEM [15].

An additional element of the study was the assessment of the model’s subjective confidence in its answers. For this purpose, after each question, the model was asked to specify its level of confidence on a five-point scale (from 1, indicating no confidence, to 5, indicating full confidence). The collected data were subjected to quantitative analysis. Microsoft Excel and Jamovi software were used for statistical analysis. The chi-squared test was used to compare the percentage of correct and incorrect answers between the question categories. The Mann-Whitney U test was used to assess the differences in the level of declared confidence between correct and incorrect answers. The threshold for statistical significance was set at p < 0.05.

To conduct an objective evaluation of the language model’s efficacy, a comparative analysis was performed, juxtaposing its performance with the results obtained by human candidates. The research material consisted of the examination paper from the PES in pediatric surgery, administered during the spring 2025 session [15].

In the second phase of the study, official aggregated statistical data concerning the outcomes of the same examination for a cohort of 19 first-time examinees were obtained [15]. These data, presented in tabular form, included key parameters such as the mean score, median, standard deviation, and the score range (minimum and maximum). The comparative analysis involved contrasting the absolute score achieved by the AI model with the distribution of scores within the group of human specialists. The pass rates for both the model and the human cohort were also assessed based on the established passing threshold of 72 points.

## Results

**Figure 1:**
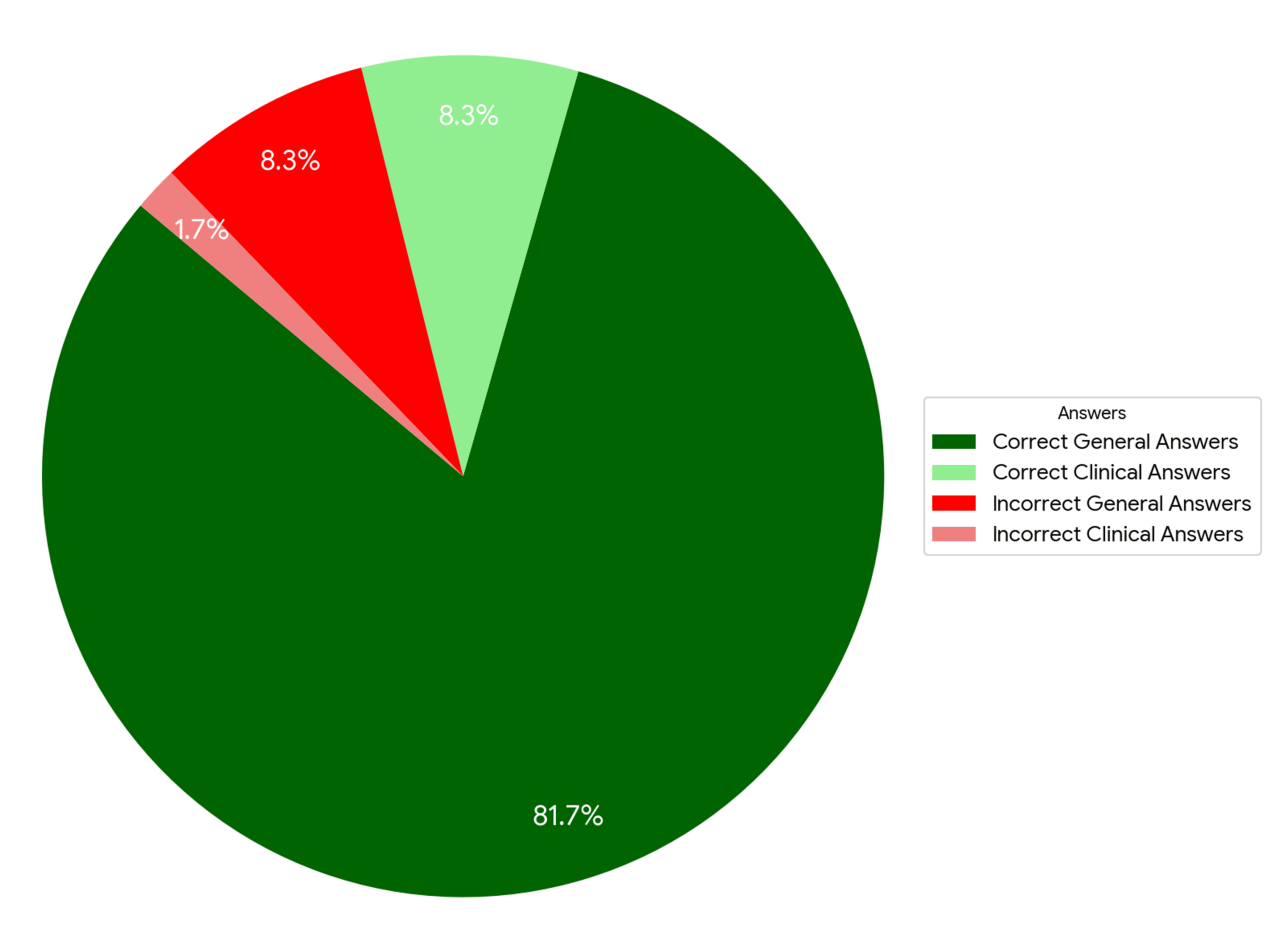
Percentage distribution of answers provided by Google Gemini 2.5 Pro.

The Google Gemini 2.5 Pro model completed the State Specialization Examination in pediatric surgery, achieving 103 correct answers out of 120 questions, which translates to an overall effectiveness of 85.83%. This result significantly exceeds the 60% threshold required to pass the exam. The distribution of correct and incorrect answers, taking into account their nature, is presented in Figure 1 and Table 1 [15].

**Table 1:** Performance of Google Gemini 2.5 Pro on the Pediatric Surgery State Specialization Examination, showing a comparison between official and model-generated answers with corresponding confidence scores. *Indicates a clinical question.

Question Number	Google Gemini 2.5 Pro Answer	Correct Answer (CEM)	Status	Confidence (1-5)
1	B	B	Compliance	5
2	D	D	Compliance	5
3	A	A	Compliance	5
4 *	C	C	Compliance	5
5	D	D	Compliance	5
6	E	E	Compliance	5
7	D	D	Compliance	5
8	A	A	Compliance	5
9	A	A	Compliance	5
10	D	D	Compliance	4
11	E	E	Compliance	5
12	B	B	Compliance	5
13	A	A	Compliance	5
14	D	D	Compliance	5
15	E	E	Compliance	5
16	D	D	Compliance	5
17	C	C	Compliance	5
18	A	A	Compliance	5
19 *	D	D	Compliance	5
20	B	B	Compliance	5
21	E	A	Non-compliance	2
22	C	C	Compliance	4
23	B	B	Compliance	5
24 *	C	C	Compliance	4
25	A	A	Compliance	5
26	C	C	Compliance	5
27	C	A	Non-compliance	1
28	E	E	Compliance	5
29	D	D	Compliance	5
30	A	A	Compliance	4
31	B	B	Compliance	5
32	A	A	Compliance	4
33 *	E	E	Compliance	5
34	B	B	Compliance	5
35	B	B	Compliance	5
36	A	A	Compliance	4
37	D	D	Compliance	4
38	A	A	Compliance	5
39	D	D	Compliance	5
40 *	D	D	Compliance	5
41	E	E	Compliance	3
42	E	E	Compliance	5
43	B	B	Compliance	4
44	B	B	Compliance	5
45	E	E	Compliance	3
46	D	D	Compliance	3
47	B	B	Compliance	5
48	B	B	Compliance	4
49	A	A	Compliance	4
50	A	A	Compliance	4
51	C	C	Compliance	4
52	C	C	Compliance	5
53	C	C	Compliance	3
54	A	A	Compliance	5
55	A	A	Compliance	3
56	A	E	Non-compliance	4
57	E	E	Compliance	5
58	D	D	Compliance	5
59	C	C	Compliance	2
60	C	D	Non-compliance	5
61	E	E	Compliance	5
62	C	C	Compliance	4
63	A	A	Compliance	5
64	A	A	Compliance	5
65	E	E	Compliance	2
66	C	C	Compliance	3
67	A	A	Non-compliance	3
68	D	D	Compliance	4
69	B	E	Non-compliance	2
70	C	C	Compliance	5
71 *	D	D	Compliance	4
72 *	B	D	Non-compliance	4
73	B	B	Compliance	5
74	D	D	Compliance	3
75	D	D	Compliance	4
76	A	A	Compliance	3
77 *	A	A	Compliance	5
78	B	B	Compliance	4
79	D	E	Non-compliance	1
80 *	C	C	Compliance	5
81	C	C	Compliance	5
82	A	A	Compliance	5
83	A	A	Compliance	4
84	B	B	Compliance	5
85	E	E	Compliance	5
86	C	C	Compliance	5
87	C	C	Non-compliance	3
88	D	C	Non-compliance	3
89	D	D	Compliance	3
90	B	B	Compliance	5
91	D	D	Compliance	4
92	B	B	Compliance	5
93	E	E	Compliance	5
94	D	A	Non-compliance	2
95	D	D	Compliance	5
96	B	B	Compliance	5
97	B	B	Compliance	5
98	A	A	Compliance	4
99	B	B	Compliance	5
100	D	D	Compliance	5
101	C	C	Compliance	4
102	A	A	Non-compliance	3
103	D	D	Compliance	3
104	A	A	Compliance	5
105 *	B	B	Compliance	5
106	E	C	Non-compliance	3
107	D	A	Non-compliance	2
108	E	E	Compliance	4
109 *	C	B	Non-compliance	3
110	E	D	Non-compliance	4
111	D	D	Compliance	5
112	C	C	Compliance	5
113	C	C	Compliance	5
114	A	A	Compliance	5
115	C	C	Compliance	4
116	A	C	Non-compliance	5
117 *	C	C	Compliance	4
118	C	C	Compliance	5
119	E	E	Compliance	5
120	D	D	Compliance	4

In a detailed analysis, the examination questions were divided into two categories: clinical and general (theoretical). In this classification, the model achieved an effectiveness of 83% for clinical questions and 91% for general questions. Despite the observed difference in results, the statistical analysis performed using the chi-squared test showed that it was not statistically significant (p = 0.417; χ² = 0.658) (Table 2).

**Table 2:** Comparison of the effectiveness of the Gemini 2.5 Pro model by question category.

Question Type	Correct Answers	Incorrect Answers	p-value	χ² value
Clinical	10 (83%)	2 (17%)	0.417	0.658
General	98 (91%)	10 (9%)		

**Figure 2:**
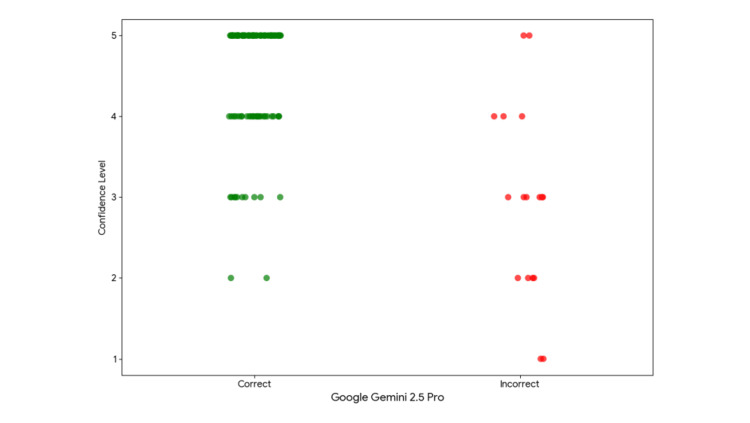
Distribution of the declared confidence levels (on a 1-5 scale) for correct and incorrect answers.

The level of confidence declared by the model for each of the provided answers was also evaluated. After performing calculations on the provided data, the Mann-Whitney U test showed that correct answers were given with a statistically significantly higher level of confidence than incorrect answers (U = 603.5, p = 0.014). The difference in the distribution of confidence levels between the two groups of answers, illustrated in Figure 2, confirms the existence of a strong correlation between the model’s self-assessment and its actual effectiveness. 

According to the official statistics, 19 candidates participated in the specialization exam during the spring 2025 session. The mean score for this cohort was 93.9 points, with a SD of 10.11. The median score was slightly higher than the mean, at 96.0 points. The highest recorded score was 106 points, while the lowest was 63. With the passing threshold set at 72 points, only one candidate (5.26%) failed the examination, indicating a very high success rate for the human cohort (Table 3) [15].

**Table 3:** Statistical outcomes of the Polish State Specialization Examination (PES) in Pediatric Surgery, administered during the spring 2025 session. The analysis pertains to a cohort of 19 first-time examinees, 18 of whom completed the test in its entirety. Source: Reference [6].

Parameter	Value
Number of examinees	19
Number of examinees with complete answers	18
Mean score	93.9
SD of scores	10.11
Median score	96
Maximum score	106
Minimum score	63
Mean difficulty index	0.772
Mean discrimination power index	0.187
Kuder-Richardson KR-20 coefficient	0.868
Test passing threshold	72
Number of examinees below the threshold	1 (5.26%)

The Google Gemini 2.5 Pro model achieved 103 correct answers on the same test, corresponding to a score of 85.83%. A comparison of this result with the statistics for the human group indicates that the AI model’s score surpassed both the mean (93.9) and the median (96.0) scores of the physicians. Concurrently, the model’s score of 103 points falls within the range of scores achieved by the human candidates, being slightly lower than the maximum score of 106 obtained by the top-performing candidate. Both the AI model and 18 out of the 19 examinees (94.74%) achieved a positive result, exceeding the required passing threshold (Table 2) [15].

The cohort demonstrated a high level of performance, achieving a mean score of 93.9 with a standard deviation of 10.11. The median score was 96.0, indicating a distribution skewed toward higher results. The range of scores spanned from a minimum of 63 to a maximum of 106. With the passing threshold established at 72 points, the success rate was exceptionally high; only a single candidate (5.26%) failed to meet this criterion [15].

Psychometric analysis of the examination revealed strong internal consistency, as evidenced by a Kuder-Richardson KR-20 coefficient of 0.868. The mean difficulty index of 0.772 suggests that the examination was of moderate to low difficulty for this particular group of candidates. Conversely, the mean discrimination power index was low at 0.187, which may indicate that the test items had limited efficacy in distinguishing between examinees with varying levels of proficiency [15].

## Discussion

The score of 85.83% obtained by the Gemini 2.5 Pro model on the State Specialization Examination in pediatric surgery exceeds the passing threshold and demonstrates the advanced capabilities of the latest generation of language models. Importantly, this result is significantly higher than the scores achieved by previous models, such as ChatGPT-4o, in examinations for other specializations, e.g., ophthalmology (78.3%) or cardiology (67.1%) [8-9]. Such a notable difference may indicate not only general technological progress in the field of AI but also the specific predispositions of the Gemini model for processing complex medical data. However, it must be emphasized that a high score on a standardized test cannot be equated with actual clinical competence. As indicated in the literature, professional examinations do not fully reflect the complexity of diagnostic and therapeutic processes, which require contextual reasoning, adaptability, and judgment under conditions of incomplete data. Moreover, evaluating LLMs using such tests carries inherent limitations due to their specific nature. On the one hand, these models are “brittle,” meaning that slight changes in the wording of a question or the order of answers can significantly affect the outcome. On the other hand, they are stochastic, meaning that repeated inquiries may produce different responses. These facts constitute a significant limitation in treating state examinations as the ultimate benchmark for artificial intelligence in medicine [16].

In the present study, no statistically significant difference was observed in the model’s effectiveness between questions requiring clinical reasoning and those verifying theoretical knowledge. This suggests that the model possesses balanced abilities in both areas, which is crucial from the perspective of medical education. An even more important finding is the statistically significant correlation between the correctness of the answer and the level of confidence declared by the model (p = 0.014). This indicates that the model exhibits a certain degree of “self-awareness” regarding the quality of the information it generates. This feature could, in the future, serve as an indicator for users, signaling when an answer requires particularly thorough human verification.

Despite the promising results, one must remember the inherent limitations of this technology. LLMs, including Gemini, are susceptible to the phenomenon of “hallucinations,” which refers to the generation of information that appears plausible but is, in fact, false [14]. Moreover, even with a positive correlation between confidence and correctness, the model can still provide an incorrect answer with maximum self-confidence, posing a serious risk in a clinical context. Therefore, the role of AI in medicine should be viewed as supportive, not as a substitute for a human expert.

The high effectiveness of the Gemini 2.5 Pro model in an examination simulation confirms its potential as an advanced tool for the postgraduate education of doctors. It can serve as an interactive review platform, an examination simulator, or a tool to help understand complex clinical concepts. Nevertheless, its integration into the medical education system requires further research and the development of ethical standards and safe implementation strategies.

The comparison of the score obtained by the Gemini 2.5 Pro model with the official examination statistics provides additional context for evaluating its capabilities. The model’s score of 103 points positions it above both the mean (93.9) and the median (96.0) achieved by the physicians who undertook the exam [15]. This suggests that the model’s performance not only meets the criteria required of a specialist but also exceeds the average level of knowledge demonstrated by the examinees. It should be noted, however, that the AI’s score, while impressive, did not surpass the maximum result achieved by the top-performing candidate (106 points) [15]. This observation reinforces the notion that while artificial intelligence is approaching expert-level proficiency and can serve as a valuable tool in educational and diagnostic contexts, the peak achievements of human specialist knowledge and analytical ability still represent the ultimate benchmark.

## Conclusions

The Google Gemini 2.5 Pro model demonstrated high effectiveness in solving questions from the State Specialization Examination in pediatric surgery, confirming the dynamic progress of AI in processing specialized medical knowledge. However, due to the study’s methodological limitations, including the small sample size and the specific nature of the test-based examination, these results should be regarded as preliminary findings for further research rather than conclusive evidence of the model’s clinical utility. The high efficacy of the Gemini 2.5 Pro model observed in this study is further supported by the fact that its score exceeded both the mean and median results of the specialist physicians. This not only confirms the model’s ability to pass a demanding examination but also highlights its potential as an advanced tool to support postgraduate medical education. Although AI demonstrates performance that surpasses that of the average specialist trainee, the higher scores achieved by physicians underscore the indispensable value of human expertise and clinical judgment in medicine.
